# Specific In Vivo Ablation of Lrig1-Positive Follicular Progenitor Cells Results in Sebaceous Gland Loss in Mice

**DOI:** 10.3390/ijms27031513

**Published:** 2026-02-03

**Authors:** Laurent Barnes, Fabienne Fontao, Evangelia Konstantinou, Jean-Hilaire Saurat, Olivier Sorg, Gürkan Kaya

**Affiliations:** 1 Department of Dermatology, University Hospital of Geneva, CH-1205 Geneva, Switzerland; 2 Department of Clinical Pharmacology and Toxicology, University of Geneva, CH-1206 Geneva, Switzerlandjean.saurat@unige.ch (J.-H.S.); olivier.sorg@unige.ch (O.S.); 3 Department of Medicine, University of Geneva, CH-1206 Geneva, Switzerland; 4 Department of Clinical Pathology, University Hospital of Geneva, CH-1205 Geneva, Switzerland

**Keywords:** Lrig1, sebaceous glands, tamoxifen, ablation, recovery

## Abstract

Leucine-rich repeats and immunoglobulin-like domains protein 1 (Lrig1) is a functional inhibitor of the epidermal growth factor receptor. Lrig1-positive stem cells are located in the isthmus region of the mouse hair follicle (HF) and are known contributors to sebaceous gland (SG) formation and homeostasis. In this study, we performed a topical tamoxifen inducible diphtheria toxin-mediated ablation of Lrig1-expressing cells in transgenic mice to investigate their function in vivo. Selective depletion of Lrig1-positive cells resulted in a complete but reversible loss of SGs, with atrophy beginning at day 14 and full recovery occurring after six months. In the absence of the Lrig1 niche, junctional-zone keratinocytes adopted an interfollicular epidermis-like phenotype (K1-positive), and repopulating cells from other epidermal compartments failed to differentiate into the sebocyte lineage. These findings demonstrate that Lrig1-positive progenitors are crucial for proper sebaceous gland morphogenesis and maintenance. Our results highlight the importance of Lrig1-positive cells in SG-related skin physiology.

## 1. Introduction

The leucine-rich repeats and immunoglobulin-like domains protein 1 (Lrig1) is one of the three members of single-transmembrane proteins. Lrig1 acts as a negative regulator of the ErbB family of receptor tyrosine kinases (RTKs), including EGFR, by enhancing its degradation, and plays an important role in epidermal homeostasis [1,2,3,4]. It also negatively regulates other RTKs, such as Met receptors. On the other hand, Lrig2 is implicated in neural development, whereas Lrig3 plays an important role in the development of the inner ear [2].

In mice, Lrig1 is expressed by epidermal progenitor cells located in the isthmus region of the hair follicle and connects the infundibulum, isthmus and the sebaceous glands (SGs) of the pilosebaceous unit. These cells, in normal conditions, feed the isthmus, the infundibulum and SGs, but in cases of injury they regenerate all the epidermal compartments, and for this reason they are considered as epidermal multipotent stem cells [2,3,5]. In adulthood, Lrig1-expressing cells only remain in the isthmus in mice. The anti-mitotic activity of Lrig1 is believed to contribute to the quiescent status of Lrig1-expressing cells [3]. It has been shown that in Lrig1-knockout mice there is an epidermal hyperplasia and a psoriasis-like phenotype. According to one study, there is no difference in the expression levels of the three LRIG members, but there is a difference in the localization of Lrig1 [6].

In human epidermis, there are clusters of Lrig1-positive keratinocytes in the basal layer on the top of the rete ridges of the interfollicular epidermis (IFE). There, neighboring Lrig1+/EGFR- and Lrig1-/EGFR+ clusters of cells can be found. Also, in human epidermis a follicular cluster of Lrig1-positive cells can be found, and they have the same actions as mouse Lrig1-positive cells, as they connect hair follicles with SGs [2,3,5]. In normal cells the expression levels of Lrig1 are low, but after specific stimulation, for example after the secretion of ligands of ErbB, these receptors are activated, and as a result Lrig1 is highly expressed in order to bind the receptors and acts as an inhibitor of epidermal growth [2,4].

Cells expressing Lrig1 generate formation during the morphogenesis of the whole pilosebaceous unit, including the SG [3,7]. More thoroughly, the SG is part of the upper pilosebaceous unit, which remains a permanent epithelium besides its gland formation, and includes the isthmus, the junctional zone and the infundibulum. In all these compartments, stem cells that secrete many factors, including Lrig1, are present [3,7,8]. In more detail, Lrig1-positive proliferative stem cells from HFs play an important role in the development of SGs (as they differentiate to sebocytes, which migrate close to IFE) as well as in the homeostasis of SGs [9,10]. These differentiated sebocytes do not proliferate; they are located in the inner compartment of the SG and produce sebum. Moreover, they express stearoyl CoA desaturase 1 (SCD1), which is an important factor for the maintenance of HFs and SGs, and they are Lrig1-negative. Furthermore, some of the cells that express SCD1 participate in the formation of two different glands, where Lrig1-negative cells are surrounded by Lrig1-positive cells [11]. During aging, the SG is characterized by hyperplasia, which is followed by atrophy and a decrease in the secretion levels of sebum. According to studies, especially in women, there is a reduction in sebum secretion after menopause [12]. The SG, even though it is a specialized gland, is still an epithelium, and so a permanent growing tissue. In different stages of life it is necessary for SGs to be replenished, and according to different studies, probably stem cells play an important role in this procedure [13]. The best characterized cells that make a significant contribution in SG homeostasis are Lrig1-positive keratinocytes from the junctional zone and from the HF. Other important cells in the renewal of sebocytes are LGR6+ progenitors, which are located above the bulge of the hair follicle, and keratin 15 (K15)+ cells [10,13,14].

We have shown that Lrig1-positive progenitor cells are the initial targets of dioxins and that topical application of 2,3,7,8-Tetrachlorodibenzo-*p*-dioxin (TCDD), which is a dioxin-like compound responsible for the induction of chloracne, can lead to the colocalization of Lrig1 and Cyp1a1 [15]. In the present preliminary study, in order to better understand the crucial role of Lrig1 in SG homeostasis, we performed topical tamoxifen inducible diphteria toxin-mediated cell ablation of Lrig1-expressing progenitor cells in mice, and we analyzed the expression of Lrig1, SCD1, keratin 1 (K1) and K15 over a six-month period of time [16,17].

## 2. Results

**Figure 1:**
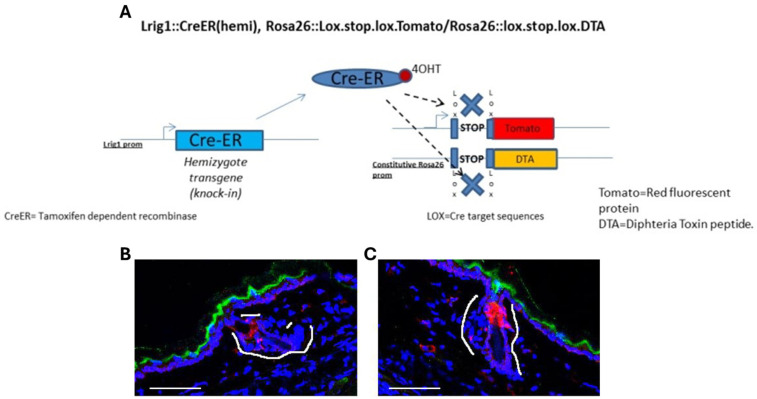
Lrig1 ablation in transgenic mice. Transgene construct and the protocol of Lrig1 in vivo ablation (**A**). Caspase 3 (AF835, R&D Systems, Minneapolis, MN) staining (red) in the isthmic area of the HF (marked by white lines) in control (**B**) and 4-OHT-treated (**C**) mouse skin (blue = DAPI). Scale bar = 87 µm.

To test the efficacy of 4-OHT treatment, we made a Caspase 3 staining on the ear samples of treated B6.129S6(Cg)Lrig1tm1.1(cre/ERT2)*R26DTA/TOM mice that showed an immediate activation of the apoptotic process in the isthmic zone of the hair follicle (Figure 1A,B). The effect of Caspase 3 was limited to the ear and no deleterious effect was observed in the tail skin, intestines or other organs of the mouse where Lrig1 is expressed, and the mice presented no general health problems.

**Figure 2:**
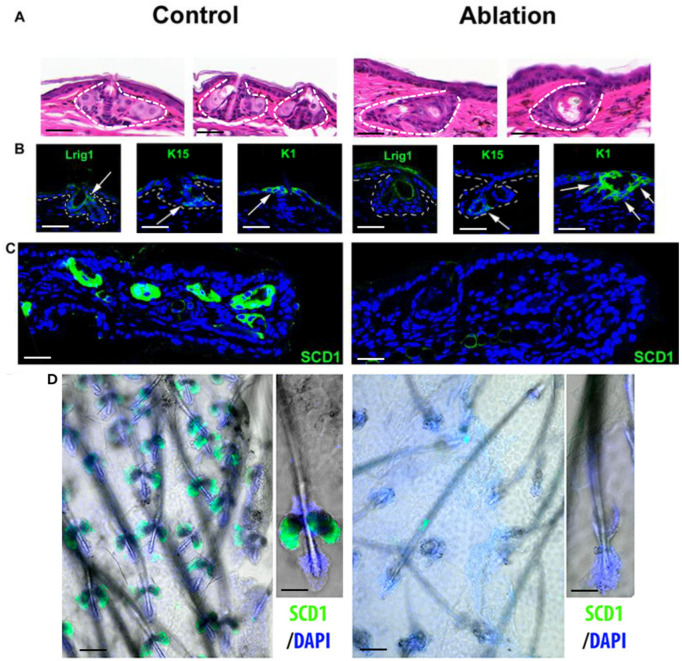
Analysis of Lrig1, SG, epidermal and follicular markers after Lrig1 ablation. H&E staining of the ear folliculosebaceous unit (FSU) in Lrig1::CreER, R26.lox.stop.lox.Tom (control) and Lrig1::CreER, R26.lox.stop.lox.Tom/R26.lox.stop.lox.DTA (ablation) transgenic mice, both treated for 14 days with a daily application of 4-OHT on the ears (**A**). Lrig1 (AF3688, R&D, Minneapolis, MN), K1 (AF109; Covance, NJ, USA) and K15 (MA1-90929, Thermo Fisher Scientific, Waltham, MA, USA) immunostainings (green) of FSU (marked by dashed lines) from the same ears ((**B**), arrows). Immunostaining of SCD1 (green, sc-515844, Santacruz Biotechnology, Dallas, TX, USA) in the ear skin of both mice (**C**). SCD1 (green) or DAPI (blue) staining of FSU of HFs in wholemount epidermis samples (**D**) (images were obtained using a Leica SP5 confocal microscope). Scale bars = 87 µm (**A**–**C**), 100 µm ((**D**), big images), 50 µm ((**D**), small images).

Staining of Lrig1 after 14 days of treatment with 4-OHT confirmed that no more Lrig1+ cells were present in the isthmic zone of the HF, where Lrig1 is usually detected (Figure 2A). K15 staining showed that the bulge remained intact in the variable part of the HF. Hair growth also seemed to be normal and no hair abnormality was observed after ablation. K1 staining indicated that the new isthmic zone expressed this differentiation marker usually expressed in the IFE (Figure 2B). Staining of the sebaceous differentiation marker SCD1 showed an almost total (95%) absence of SGs (Figure 2C). Analysis of hair follicles in the wholemount epidermis samples confirmed the absence of SGs (Figure 2D).

**Figure 3:**
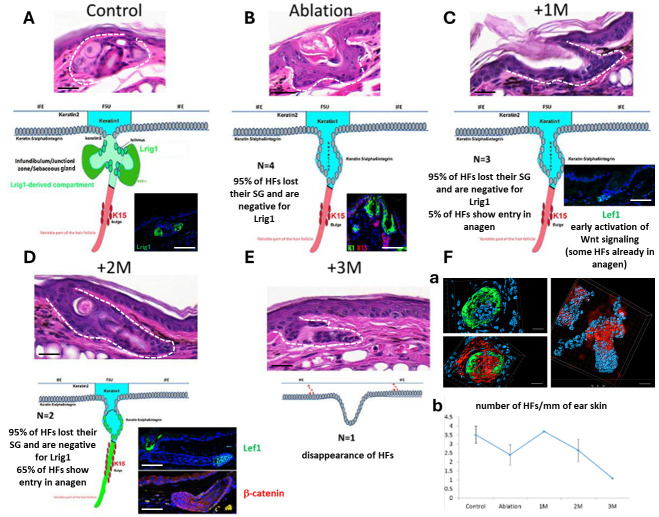
Analysis of FSU in mouse ear skin after Lrig1 ablation at different time points. H&E staining of the ear FSU (marked by dashed lines) in Lrig1::CreER, R26.lox.stop.lox.Tom (control) (**A**) and Lrig1::CreER, R26.lox.stop.lox.Tom/R26.lox.stop.lox.DTA (ablation) (**B**) transgenic mice, both treated 14 days with a daily application of 4-OHT on the ears, and 1 (**C**), 2 (**D**) and 3 (**E**) months later. Schematic representations of FSU are shown as lower images in A, B, C, D and E (normal expression localizations of Lrig1, K1 and K15 in (**A**)). Lrig1, K1, Lef1 (green, C12A5, Cell Signaling, Danvers, MA, USA) and K15, β-catenin (red, non-phosphorylated form, D10A8 XP, Cell Signaling, Danvers, MA, USA) immunostainings of FSU are shown in (**A**–**D**). SCD1 (green), tomato (red) or DAPI (blue) staining of FSU of HFs in wholemount epidermis samples in (**F**,**a**) (upper left: control mice with SGs; lower left: control mice with tomato after 1 month; right: Lrig1-ablated mice after 2 months with elongated anagen HF; images were obtained using Imaris software). Number of HFs in Lrig1-ablated mice is shown in (**F**,**b**). Scale bars = 87 µm (**A**–**D**), 10 µm (**F**,**a**). M = month(s), N = number of mice used.

One month after treatment with 4-OHT, 95% of the HFs with loss of SGs were negative for Lrig1 and 5% of the HFs showed signs of entry into the anagen phase with early activation of the Wnt signaling pathway (Lef1 positivity) (Figure 3A–C). Two months after treatment, SGs were still absent in 95% of the HFs, whereas 65% of the HFs entered into the anagen phase as shown by the activation of the Wnt pathway (Lef1 and β-catenin immunostainings) (Figure 3D) and the confocal microscopy (Figure 3Fa). Three months after treatment, most of the HFs disappeared (Figure 3E,Fb), and this hair loss was macroscopically visible on the ear skin, but reappeared after 6 months (macroscopic observation, quantitative data not available).

**Figure 4:**
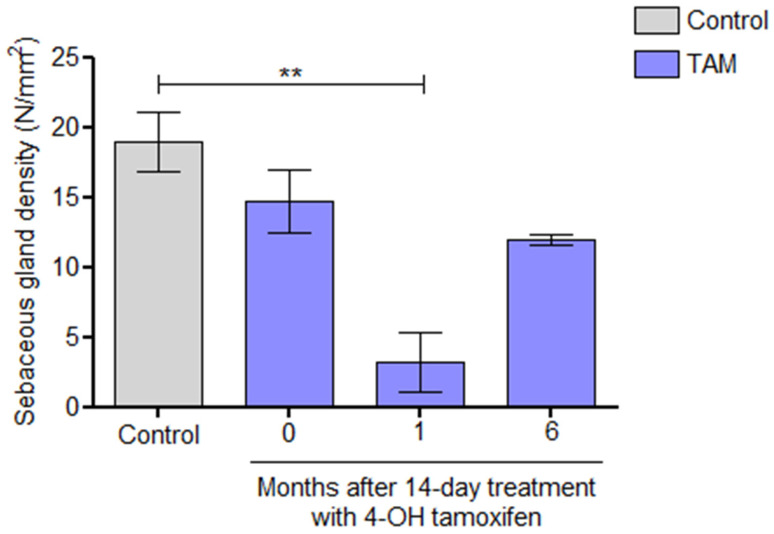
SG recovery following Lrig1 ablation. The total area was determined (in mm^2^) for the ear section, all sebaceous glands in this area were counted, and finally the number of SG/mm^2^ was calculated. The quantification of SGs for each group was performed at 4 high-power field (HPF) corresponding to a 1 mm^2^ area per subject (10× eyepiece with a field number of 22 and a 40× objective lens: HPF diameter: 0.55 mm and HPF area: 0.237 mm^2^) (control = no treatment, TAM = mice treated with 4-OHT). Statistical analysis was performed by ANOVA (** *p* < 0.01).

Analysis of the density of SGs in ear skin sections at different time points showed the recovery of SGs after six months (Figure 4).

The SG density decrease was at a maximum one month following 4-OHT treatment and started to recover progressively from the second month after the application of 4-OHT, reaching the initial density levels after 6 months. This kinetics of SG clearance and recovery was very similar to that observed after a topical treatment on mouse ears with the AhR agonist TCDD [15].

**Figure 5:**
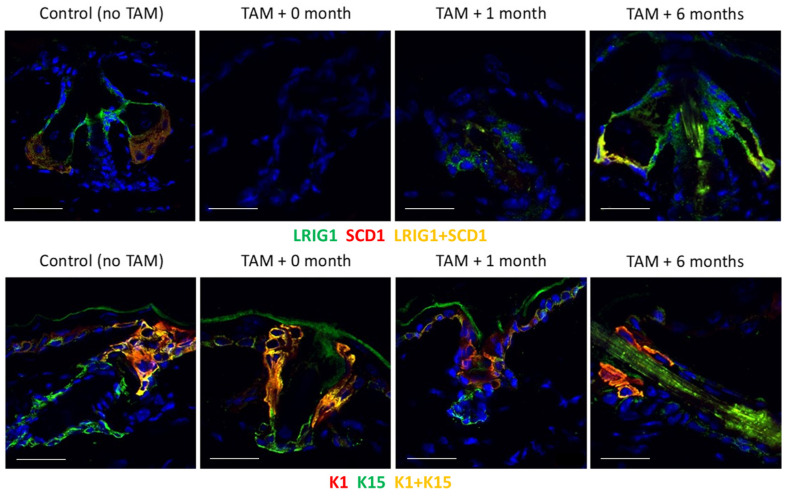
SG and epidermal markers in mouse skin following Lrig1 ablation. Staining: Lrig1 (green), SCD1 (red), merge Lrig1 + SCD1 in yellow, K1 (red), K15 (green), merge K1 + K15 in yellow. Immunostaining studies were realized on the cryo-sections of mouse epidermis that were fixed in formalin before incubating with the respective antibodies. These sections were embedded in OCT^®^ (Sakura, Japan) cryopreservative medium before making cryosections. Cryosections were mounted in a Dapifluoromount-G^®^ (Southerntech, OK, USA). Slides were treated with citric acid (10 mM, pH6) for antigen retrieval. Scale bar = 20μm.

To examine the possible role of Lrig1 ablation in the homeostasis of SGs, we analyzed the expressions of Lrig1, SG marker SCD1 [18], epidermal marker K1 and HF bulge region marker K15 [19] at various time points following the ablation of Lrig1 cells with 4-OHT in mice bearing the Lrig1::CreERT2 and R26::DTA transgenes (Figure 5).

We found that Lrig1 and SCD1 were reduced 1 month after selective ablation, and completely recovered 6 months later (Figure 5). After ablation, K1-positive cells from the infundibulum filled the isthmus and became adjacent to K15-positive cells from the bulge region (Figure 3 and Figure 5).

Despite the changes described for the SGs and HFs with entry into the anagen phase and the disappearance of HFs, no hair loss or hair overgrowth was observed in the ear skin of these mice morphologically. As the application of 4-OHT was performed only on the ear, the back skin HFs were not analyzed.

## 3. Discussion

It is known that Lrig1 is a transmembrane protein that is expressed by epidermal progenitor cells and acts as a negative regulator of EGFR as it antagonizes its ligand. Furthermore, Lrig1-positive cells participate in the maintenance of the parts that are localized in the upper pilosebaceous unit, including SGs and the infundibulum [1]. As mentioned in the literature, Lrig1-positive cells give rise to sebocytes and they are one of the different cell types that play an important role in their renewal and contribute to the homeostasis of SGs [2].

In the current study, we observed that in the absence of the Lrig1-positive niche, keratinocytes of the isthmus/junctional zone differentiated into interfollicular epidermis-like keratinocytes. Following Lrig1 ablation, cells from other epidermal compartments rapidly repopulated the junctional zone and the pilosebaceous space. However, they expressed K1, a marker of interfollicular epidermis, suggesting that the Lrig1-positive niche is probably crucial for SG maintenance, and that cells that repopulated the niche were not able to differentiate into the sebocyte lineage. Our results show that the Lrig1-positive progenitor cell compartment may play an important role in SG-related skin pathologies. We also demonstrated that the selective ablation of Lrig1-positive cells induces the disappearance of SGs and their markers. Although Lrig1 expression is largely restricted to the upper HF, particularly the isthmus and junctional zone, this apparent discrepancy suggests that Lrig1-positive cells represent a critical progenitor population for SG maintenance rather than marking differentiated sebocytes. Lineage commitment from Lrig1-positive cells may occur without sustained Lrig1 expression, such that their differentiated progeny populate the SG while remaining Lrig1-negative. Alternatively, Lrig1-positive cells may exert non-cell-autonomous effects by providing essential niche signals required for SG renewal or survival. Loss of this regulatory population could therefore indirectly destabilize the sebaceous lineage. Together, these findings indicate that the spatial restriction of Lrig1 expression does not preclude a broader functional role in SG homeostasis and underscore the importance of upper HF progenitors in maintaining SG integrity. Moreover, the clearance of SGs is reversible, and the number of SGs recovered six months after the ablation of Lrig1. The delayed regeneration of SGs following Lrig1-positive cell ablation suggests the activation of compensatory stem cell plasticity within the HF epithelium. One hypothesis is that alternative progenitor populations, including bulge-derived stem cells that are normally restricted to HF lineages, can be mobilized to replenish the SG in response to loss of the Lrig1-positive compartment. Such lineage reprogramming may be facilitated by changes in the local signaling environment, including activation of FGFR2 signaling, which has been implicated in SG development and sebocyte differentiation (13). In parallel, infundibular or junctional zone keratinocytes may contribute through fate flexibility under conditions of niche vacancy. These compensatory mechanisms may operate through modulation of Wnt/β-catenin, EGFR, and PPARγ pathways to restore sebaceous identity. Findings from another study demonstrated that deletion of Scd1 in Lrig1-positive cells, apart from disruption of SGs, also causes progressive hair loss and the appearance of dysmorphic HFs. Moreover, there was a disruption in autocrine Wnt signaling and paracrine Hedgehog signaling due to Scd1 deletion. Changes in these signaling pathways resulted in degeneration of the dermal papilla and impaired matrix progenitor cell proliferation. Overall, data from this study suggest that Lrig1-positive cells are important not only for SG maintenance, but also for the mediation of HF homeostasis through signaling pathways that are Scd1-dependent [20]. In our study, we did not observe hair loss or dysmorphic HFs; however, we noted the presence of a substantial amount of HFs entering into the anagen phase shown by Wnt pathway activation as a result of β-catenin and Lef1 expression, starting from 1 month after Lrig1 ablation. Apparently, the loss of Lrig1-positive cells induces this anagen activation by an unknown signal, but with no morphological change in the ear skin hair. The altered anagen activation observed following Lrig1-positive cell ablation suggests that upper HF progenitors and SG integrity contribute to the regulation of hair cycling. One potential mechanism is that Lrig1-positive cells modulate signaling pathways known to influence anagen entry, including EGFR, Wnt/β-catenin and FGF signaling. As Lrig1 functions as a negative regulator of EGFR activity, its loss may result in aberrant EGFR signaling that perturbs the balance between quiescence and activation within the follicle. In addition, disruption of the SG may indirectly affect hair cycling by altering the local lipid milieu or depriving the follicle of sebocyte-derived paracrine factors that have been implicated in maintaining follicular stem cell homeostasis. SGs have also been proposed to act as a structural and signaling interface between the interfollicular epidermis and the hair follicle; their loss could therefore destabilize niche interactions required for coordinated hair cycle progression. Finally, ablation of Lrig1-positive progenitors may provoke inflammatory or stress responses within the skin, which are known to influence anagen onset.

In a previous study, we showed that the topical application of TCDD on mouse ears induced a reversible atrophy of SGs [15]. The similarity between the kinetics of clearance and recovery of SGs following their suppression by TCDD or Lrig1 ablation led us to consider that one of the initial skin targets of TCDD might be progenitor Lrig1 cells, which colocalize with CYP1A1 (Supplementary Material Figure S1). Moreover, we found that the downregulation of Lrig1 in cultured cells and the subsequent application of TCDD reduced the expression and activity of CYP1A1 [15].

It seems that TCDD interferes with the regulation of gene expression in these cells, which no longer migrate and differentiate to sebocytes, but produce so-called MADISH (metabolizing acquired dioxin-induced skin hamartoma) skin lesions with the loss of SGs, the cutaneous hallmarks of dioxin exposure in humans [21].

## 4. Materials and Methods

Transgenic B6.129S6(Cg)Lrig1tm1.1(cre/ERT2)*R26DTA/TOM mice (bearing the Lrig1::CreERT2 and R26::DTA transgenes; Jackson Laboratory, Bar Harbor, ME, USA) and non-transgenic control mice were used in this study. In more detail, the mice that were used in the current study were heterozygotes with a wild-type gene and another gene with the Cre-ER (tamoxifen dependent recombinase) inserted in the Lrig1 exon, right in front of the Lrig1 promoter. As for the Rosa26 locus, it had a constitutively activated promoter. Downstream of this promoter, some animals had either the tomato (TOM) transgene inserted, or DTA (diphtheria toxin fragment A). The expression of both genes was prevented by a stop codon flanked by 2 lox-sequences (target of the CRE). After the application of the 4-hydroxytamoxifen (4-OHT) (Sigma, Saint Louis, MI, USA), the 4-OHT bound to the Cre (which is expressed only in cells expressing Lrig1) and so translocated to the nucleus. Once in the nucleus, the Cre removed the stop codon of tomato/DTA so these proteins could be expressed. Cells then became red and started expressing DTA. The latter was toxic and induced the apoptosis of the given cell (Figure 1).

Using the above-mentioned conditional gene modification technology [22], we induced a selective suppression of epidermal cells expressing the Lrig1 gene in the adult mice bearing the Lrig1::CreERT2 and R26::DTA transgenes following topical application of 5μΜ 4-OHT once per day, on the ear of each mouse, for 14 days. The SG density was determined in ear skin sections with H/E staining for each time point.

For the detection of SG markers Lrig1 and SCD1 and the epidermal markers K1 and K15, ear skin or wholemount epidermis samples were analyzed with immunofluorescence at various time points following Lrig1 ablation.

## 5. Conclusions

In this preliminary study with a limited number of mice studied, the following conclusions can be made.

- The Lrig1 cluster was effectively removed by the tamoxifen-dependent DTA activity in Lrig1::CreER, R26.lox.stop.lox.Tom/R26.lox.stop.lox.DTA transgenic mouse ear skin.
- The basal keratinocytes located in the infundibulum (and negative for Lrig1) seemed to repair the FSU. The new constant part of the FSU became entirely positive for K1 in the differentiated layers.
- The SGs disappeared, but were recovered after 6 months, suggesting that the Lrig1+ compartment is not the only stem cell reservoir able to produce SG lineage keratinocytes.
- Lrig1-positive stem cells/isthmus are dispensable for the homeostasis of the IFE and the variable part of the hair follicle (bulge dependent part), at least in homeostatic conditions.
- Lrig1 ablation induces an anagen phase shift in the HFs and Wnt pathway activation 1 month after the ablation with disappearance of the hair follicles after 3 months, which are, as are SGs, recovered after 6 months.
- Topical application of TCDD on mouse ears induced a reversible atrophy of SGs, suggesting that the initial skin targets of TCDD might be Lrig1-positive cells, which colocalize with CYP1A1.
- Further studies are needed to understand the molecular mechanisms of SG loss induced by Lrig1-positive cell ablation and the role of Lrig1 in SG homeostasis.

## Data Availability

The original contributions presented in this study are included in the article. Further inquiries can be directed to the corresponding author.

## Supplementary Materials

The following supporting information can be downloaded at: https://www.mdpi.com/article/10.3390/ijms27031513/s1.
