# Supplementary material for: Specific In Vivo Ablation of Lrig1-Positive Follicular Progenitor Cells Results in Sebaceous Gland Loss in Mice
Source: Int J Mol Sci. 2026 Feb 3;27(3):1513. doi: 10.3390/ijms27031513 (PMC12897776; doi:10.3390/ijms27031513)
Supplement: Supplementary file 1 [file ijms-27-01513-s001.zip › ijms-4023121-supplementary.pdf]

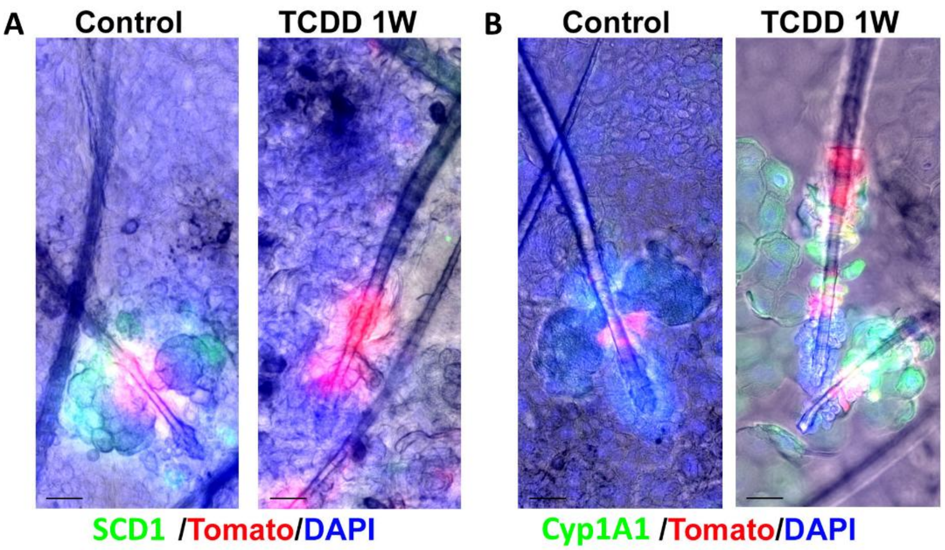

Supplemental Figure S1. Analysis of SGs in the ear skin of Lrig1::CreER, R26.lox.stop.lox.Tom mice treated with TCDD and injected with Tamoxifen (intraperitoneal injection 2 × 2 mg; T5648, Sigma Saint Louis, MI)). SCD1 (green) (A), tomato (red) (A, B), CYP1A1 (green) (B) or DAPI (blue) (A, B) staining of FSU of HFs in wholemount epidermis samples in control and TCDD-treated mice (A, B) (images were obtained using a Leica SP5 confocal microscope). Note the presence of SCD1-positive SGs in control mice (A) with loss of them in TCDD-treated mice after 1 week (A) and the presence of tomato (Lrig1) in the isthmus region of the HF (A, B) and its colocalization with CYP1A1 (B). 1W= 1 week. Scale bar = 50 µm.
